# Cognitive Behavioral Therapy for Insomnia: A Promising Treatment for Insomnia, Pain, and Depression in Patients With IBD

**DOI:** 10.1093/crocol/otaa052

**Published:** 2020-06-24

**Authors:** Jessica K Salwen-Deremer, Corey A Siegel, Michael T Smith

**Affiliations:** 1 Department of Psychiatry, Dartmouth-Hitchcock Medical Center, Lebanon, New Hampshire, USA; 2 Section of Gastroenterology and Hepatology, Department of Medicine, Dartmouth-Hitchcock Medical Center, Lebanon, New Hampshire, USA; 3 Department of Psychiatry, The Johns Hopkins School of Medicine, Baltimore, Maryland, USA; 4 Department of Nursing, The Johns Hopkins School of Medicine, Baltimore, Maryland, USA; 5 Department of Neurology, The Johns Hopkins School of Medicine, Baltimore, Maryland, USA

**Keywords:** IBD, sleep disturbance, insomnia, CBT-I, chronic pain, depression

## Abstract

Over 75% of people with active inflammatory bowel diseases (IBDs) report sleep disturbances, which heighten risk for IBD relapse and flares. Despite mounting evidence for sleep disturbances in IBD, discussion of treatment is severely limited. The most common sleep disturbance, insomnia, occurs in over 50% of adults with chronic health conditions. Herein we describe the gold standard treatment for insomnia, Cognitive Behavioral Therapy for Insomnia (CBT-I). Although yet to be studied in IBD, CBT-I reduces a number of IBD-related comorbidities, including chronic pain, depression, and systemic inflammation. We describe treatment with CBT-I, the impact of CBT-I on these comorbidities, and recommendations for providers.

## INTRODUCTION

Inflammatory bowel disease (IBD) refers primarily to Crohn disease (CD) and ulcerative colitis (UC)—chronic inflammatory diseases of the gastrointestinal tract that affect over 1.2 million Americans,^1^ though prevalence is increasing.^2^ Symptom flares involve difficult and painful bowel symptoms and can result in missed work, emergency room visits, lengthy hospitalizations, and life-altering surgeries.^3,4^ Flares are also associated with increased systemic inflammation,^5^ which likely worsens and maintains comorbid non-gastrointestinal symptoms, such as insomnia, widespread pain, and ocular, rheumatologic, and dermatologic manifestations of IBD.^6–9^

Unfortunately, even when IBD disease activity is well managed with medication and lifestyle modification, patients continue to report chronic pain and fatigue, and have a 2-fold heightened risk for depression.^10–12^ Pain is present in over 70% of individuals with IBD^10,13^ and is associated with decreased quality of life and greater disease severity.^13^ It is thought of as an “alarm symptom” in IBD, and can lead to increased medication use and other heightened levels of care.^14^ Pain can also result in increased narcotic use, leading to increased complications and consequences.^15,16^ Depression is present in 21.2% of individuals with IBD and 34.7% of those with active disease.^12^ It is linked with increased disease activity,^17^ decreased quality of life, and predicts future risk for relapses and the need for surgery and/or hospitalization.^18–20^ As is described in greater detail below, insomnia is an independent risk factor for pain chronification, depression, and increased systemic inflammation. Given that insomnia is highly modifiable, treating insomnia has promising IBD disease management implications that require investigation.

Over 75% of individuals with active IBD (including both CD and UC) also suffer from sleep disturbance,^21,22^ and prospective studies demonstrate that sleep disturbance predicts greater likelihood of symptom flares.^21,23^ Emerging research utilizing objective sleep measures, such as actigraphy, indicates that individuals with at least moderately severe CD experience more sleep continuity disturbances than individuals with mild or remissive disease, including both minutes awake after sleep onset and sleep efficiency.^24^ Subjectively, patients also report sleep disturbance to be the most important factor driving IBD-related poor quality of life^25,26^ and believe that poor sleep contributes to next-day gastrointestinal symptoms.^26,27^ Further supporting the relationship between sleep disturbance and disease activity, recent prospective research indicates that both vedolizumab and anti-tumor necrosis factor (TNF) biologic therapies were associated with improvements in sleep, and these improvements were sustained for at least 1 year.^28^ These researchers also found that sleep disturbances were associated with increased disease activity, increased depression, and use of opioids.

This relationship between sleep disturbance and IBD is likely bidirectional, and may be mediated at least in part by inflammation. Experimental studies of sleep deprivation demonstrate that sleep disturbance results in increases in inflammatory markers associated with IBD, such as interleukin-6 (IL-6), TNF-α, and C-reactive protein (CRP).^29,30^ Conversely, research also indicates that sleep parameters respond to immune system challenges (eg, endotoxin injection and IL-6 injection).^29^ These bidirectional relationships likely lead to a cycle wherein sleep disturbance and IBD worsen and maintain one another, deteriorating quality of life and worsening overall disease activity and inflammation.^31^

Sleep disturbances have also been demonstrated to be important in other inflammatory disorders. For example, in individuals with rheumatoid arthritis, laboratory-induced partial sleep deprivation led to greater increases in fatigue, depression, anxiety, pain severity, and number of painful joints compared to healthy controls.^32^ Pilot research in patients with rheumatoid arthritis also indicates that in patients with sleep disturbances, administration of the IL-6 receptor antagonist tocilizumab may lead to significant improvements in sleep quality and daytime sleepiness, in addition to overall improvements in disease severity.^33^ Similarly, these patterns of sleep disturbance are also seen in other inflammatory disorders such as systemic lupus erythematosus and inflammatory skin disorders.^34,35^

At present, the literature on sleep disturbances in IBD lacks systematic investigation with objective measures and is predominantly limited to questionnaire based studies of general sleep complaints,^31,36^ which provide limited information on specific sleep characteristics or disorders linked with IBD. Prior reviews on sleep and IBD have generally focused the role of sleep in inflammation with brief commentary and limited rationale on the potential importance of treating sleep disorders in IBD.^6^ None of the extant literature discusses specific treatment recommendations. With the overall aim to stimulate increased clinical understanding of and research on sleep in IBD, this topical review focuses on insomnia disorder. Specifically, we provide empirical support for why Cognitive Behavioral Therapy for Insomnia (CBT-I), the recommended first line treatment for chronic insomnia disorder, may be particularly efficacious and important in managing insomnia and comorbid pain and depression in IBD.

## SLEEP DISTURBANCE, INSOMNIA, AND INTERVENTION

While poor sleep can be attributed to a variety of different sleep disorders and environmental factors, insomnia disorder is characterized by difficulty falling asleep, staying asleep, or early morning awakening, despite adequate sleep opportunity that occurs at least 3× per week for at least 3 months, and which results in daytime distress or impairment.^37^ Although insomnia disorder is often comorbid with psychiatric disorders, substance use disorders, and other sleep disorders, such as sleep apnea, diagnosis of insomnia requires that it cannot be fully explained by or occur exclusively during the course of these disorders. This comorbidity highlights the importance of a thorough diagnostic evaluation in patients presenting with insomnia symptoms. For information on assessing, evaluating, and diagnosing insomnia, please see 2020 chapter by Ong et al.^38^

Insomnia disorder has a general population prevalence of 10%^39,40^ and over 50% in chronic medical^41^ and psychiatric disorders^42^ conditions, with even higher estimates in chronic pain populations.^43,44^ To the best of our knowledge, there are currently no published studies that estimate the prevalence of insomnia in IBD. Case–control studies demonstrate heightened sleep continuity problems in IBD. For example, 71% of participants with IBD compared to 13% of controls took longer than 30 minutes to fall asleep at least once per week,^27^ suggesting potentially high rates of sleep onset insomnia in IBD.

Cognitive Behavioral Therapy for Insomnia is the recommended first line treatment for chronic insomnia by the American College of Physicians,^45^ due to its comparable effectiveness, enhanced durability, and side-effect profile compared to pharmacotherapy.^46,47^ CBT-I has been shown to be broadly effective in patients with^48,49^ and without^50^ medical and psychiatric comorbidities, as well as in older adult populations.^51^ Gold standard CBT-I involves multiple intervention components and typically consists of 4–8 visits with a sleep psychologist or similarly trained provider, board certified Behavioral Sleep Medicine. Standard components include sleep restriction therapy, stimulus control, relaxation training, cognitive therapy, and sleep hygiene education.^52^

Sleep restriction, stimulus control, and sleep hygiene are all specific to CBT-I, whereas relaxation training and cognitive therapy also appear in Cognitive Behavioral Therapy (CBT) for other problems, such as anxiety or depression. Sleep restriction involves reducing one’s time in bed (the sleep opportunity) in order to consolidate sleep, improve sleep efficiency, and improve sleep drive.^53^ Stimulus control is based on classical conditioning, and reestablishes and strengthens the bed as a cue for sleep as opposed to other physiologically arousing activities (eg, watching TV, planning, and worrying).^54^ Sleep hygiene involves adjusting behavioral, environmental, and other sleep-related factors that may interfere with sleep (eg, drinking caffeine late in the day; sleeping in a too hot or too bright bedroom).^55^ These 3 components address *perpetuating* factors in chronic insomnia, as described by Spielman’s 3P model, wherein acute insomnia becomes chronic based on predisposing (eg, hyperreactivity; altered circadian rhythms), precipitating (eg, development or exacerbation of a medical problem), and perpetuating factors (eg, excess time spent in bed trying to rest).^56^ With the exception of sleep hygiene education, most of the individual intervention components have been found to be effective monotherapies^57,58^ with the strongest evidence and more robust treatment effects attributed to a multicomponent CBT-I.^59^ It is important to highlight that basic sleep hygiene education on its own has not been found to be effective,^42,60^ and thus is often used as a control condition for randomized controlled trials.^61,62^

Remission rates for insomnia following CBT-I are typically between 65% and 70%,^59^ and improvement of sleep continuity parameters is similar across number of visits and whether CBT-I is delivered individually or in a group.^50,63,64^ Importantly, CBT-I is not only effective in improving a variety of sleep disturbances, it has also demonstrated effectiveness in reducing comorbidities and residual symptoms that occur in people with IBD, including pain, depression, and inflammation.

## CBT-I AND PAIN

Across medical populations, chronic pain and insomnia are often comorbid,^65^ and insomnia is the most common sleep disorder in patients with chronic pain.^66^ Longitudinal data indicate that although pain disrupts sleep, insomnia is an important driver of pain symptoms across chronic pain conditions, likely via direct alterations in central pain processing circuits that increases pain sensitivity.^65,67,68^ Based on these established relationships, it is unsurprising that CBT-I can result in improvements in pain. A 2015 meta-analysis of nonpharmacological treatments of insomnia in patients with chronic pain^69^ demonstrated large improvements in sleep quality, moderate improvements in fatigue, and small, but clinically significant reductions in pain. Individual studies have demonstrated more specific effects and new studies are identifying ways to optimize the analgesic benefits of CBT-I.

For example, 2 large randomized controlled trials of CBT-I compared to active control in patients with insomnia and osteoarthritis^70,71^ have demonstrated that CBT-I not only results in large improvements in primary sleep outcomes, but also improves pain at long-term follow-up. In one of these studies,^70^ across both CBT-I and active control (a validated placebo condition—behavioral desensitization), a third of all participants reported at least 30% reduction in clinical pain at 6 months. Secondary analyses of these data^72^ also showed that attaining about 6.5 hours of sleep by mid-treatment was particularly important for improvement in pain at 6 months. A similar, larger trial by one of these groups^73^ demonstrated that CBT for pain and insomnia resulted in better 18-month pain reductions than CBT for pain alone in participants who had more severe pain and insomnia at baseline.

Similarly, a number of randomized controlled trials have also evaluated the impact of CBT-I on fibromyalgia pain. In one of these studies,^74^ CBT for pain was compared with a combined treatment of CBT for both pain and insomnia. Results indicated that both of these treatments led to significant improvements in impact of pain and pain coping compared to waitlist control. However, only the treatment that included insomnia resulted in improvements in pain intensity. In another study by this same research group,^61^ CBT-I resulted in improvements in pain intensity in 50% of participants, compared to 31% of participants in active control (sleep hygiene).

Finally, in addition to improving sleep and pain severity, CBT-I may result in improvements in other aspects of chronic pain, including reductions in pain interference, pain catastrophizing, and pain-related disability, and improvements in pain coping.^61,75,76^ Particularly relevant to individuals with IBD, research indicates that following either CBT-I or a waitlist control for comorbid osteoarthritis and insomnia, participants whose insomnia had improved evidenced significantly lower levels of IL-6 before and during experimental pain testing.^77^ Further, participants with larger decreases in mean IL-6 from baseline to follow-up also had greater improvement in clinical pain. Participants with improved insomnia also demonstrated attenuated IL-6 and TNF-α responses to an evoked laboratory pain challenge. Other studies have demonstrated that compared to healthy controls, primary insomnia is associated with increased IL-6 inflammatory reactivity to laboratory evoked pain.^78^

## CBT-I AND DEPRESSION

Sleep disturbance, including both increased sleep/hypersomnia and difficulty sleeping/insufficient sleep, is a core symptom of depression,^37^ and ~90% of individuals with depression report some difficulty with or changes in their sleep patterns.^79^ Sleep disturbance is also predictive of both initial episodes of major depressive disorder (MDD)^80,81^ and of future relapses.^82,83^ Thus, it is unsurprising that both a recent meta-analysis and a recent systematic review found CBT-I not only to be effective in treating insomnia in people with depression, but also reducing depression in people with comorbid depressive disorders and insomnia.^84,85^ Individually delivered CBT-I was also found to be associated with moderate improvement in both depressive symptoms and fatigue at posttreatment, with medium effect sizes for improvement in both (Cohen *d* = .46 and .45, respectively).^84^ However, results indicated that study findings were heterogeneous, particularly with regard to findings on fatigue, and thus should be interpreted with caution.

These results are evident in a number of recent trials evaluating the impact of CBT-I compared to different kinds of control groups and across different depressive populations. In a smaller study (n = 30) comparing escitalopram to CBT-I plus escitalopram or to control in patients with both MDD and insomnia,^86^ authors found that the CBT-I group evidenced the most overall improvement. More specifically, the addition of CBT-I to escitalopram led to higher rates of remission of depression (61% vs 33.3%) and insomnia (50% vs 7.7%). Another group compared CBT-I to escitalopram + active control (sleep hygiene) in patients with MDD and insomnia (n = 107).^87^ They found that all patients improved significantly in depressive symptoms, and while patients receiving CBT-I improved on objective measurements of sleep, the escitalopram only group demonstrated worsened sleep. Importantly, CBT-I delivered remotely (as compared to in-person) has also been demonstrated to be effective. Recently, van der Zweerde et al compared online CBT-I to control (sleep diary only)^88^ and found that CBT-I produced significant improvements in both depression (medium to large effect; Cohen *d* = .76) and insomnia (large effect; Cohen *d* = 2.36), reporting that the magnitude of the improvements in depression was comparable to improvements found in in-person, depression-specific CBT. These results were maintained at 3- and 6-month follow-up. Thus, similar to findings in the pain literature described above, CBT-I can result in significant improvements in clinical depression.

## CBT-I AND INFLAMMATION

Mechanistic studies demonstrating how improving insomnia alters pain processing and depression pathophysiology are ongoing, but 1 common pathway may be the insomnia and sleep disruption links with heightened inflammation.^65,89–91^ Irwin et al^92^ recently performed a meta-analysis of 72 studies that evaluated the relationship between CRP, IL-6, and/or TNF-α and sleep disturbances. Results indicated that sleep disturbance was associated with higher levels of IL-6 and CRP and that short sleep duration, a common consequence of insomnia, was associated with higher levels of CRP. In healthy volunteers, sleep restriction increased levels of IL-6, and these elevations were associated with increased pain that could not be explained by fatigue.^89^ Similarly, nocturnal IL-6 secretion is significantly greater in people with insomnia compared to age- and gender-matched controls and is associated with increased time spent awake and decreased time spent in slow wave sleep.^93^

These same inflammatory markers have also been evaluated more broadly in depression treatment. In a recent systematic review on CBT for depression and inflammation,^94^ Lopresti et al found that CRP, IL-6, and TNF-α were most commonly assessed. Of 23 studies evaluated, 14 reported reduction in at least 1 inflammatory marker and 3 studies demonstrated poorer treatment responses in participants with high baseline inflammation. In one pilot study^95^ of women with a first episode, treatment with CBT for depression resulted not only in improvements in depressive symptoms, but also a mean decrease of IL-6 by 33%. Reductions in IL-6 were also associated with decreases in depressive symptoms. Similarly, a larger randomized controlled trial of CBT vs an active control^96^ found that CBT for depression resulted in not only a significant reduction in depressive symptoms, but also a reduction in IL-6 and TNF-α. Combined, these studies suggest that improvement in depressive symptoms may be associated with a reduction in inflammatory cytokines.

CBT-I has also been specifically linked to improvements in a number of inflammatory cytokines. In one of the largest randomized controlled trials evaluating the impact of CBT-I on insomnia and inflammation,^97^ results indicated that CBT-I was associated with a reduced risk of high CRP levels (>3.0 mg/L) at 16 months as compared to both tai chi and a sleep education control. Remission of insomnia was also associated with lower levels of CRP at 16 months. In another study with this same sample,^98^ CBT-I was associated with mid-treatment reductions in intracellular measures of inflammation, including stimulated monocytic production of IL-6 and TNF-α.

## CLINICAL IMPLICATIONS

For IBD providers, screening for sleep disorders and insomnia is recommended. While a full sleep assessment is not possible, providers may ask patients general questions about sleep habits or use a well validated screening tools, such as the Insomnia Severity Index^99^ to consider whether a referral for a sleep specialist evaluation is warranted. Patients reporting trouble initiating or maintaining sleep, 3 or more days per week for 3 months or more should be referred for an insomnia evaluation and consideration for CBT-I by sleep medicine or a qualified behavioral sleep medicine provider.

However, CBT-I is an underutilized intervention^100^ and is not always widely available. If patients are unwilling or unable to seek in person CBT-I, consideration can be given to recommending evidence based digital CBT-I approaches, such as Sleepio.com^101^ or Somryst,^102^ which was recently approved by the Food and Drug Administration as a medical device to treat insomnia. Telemedicine-based CBT-I may also provide a unique opportunity to connect people with insomnia to specialists, and it is as effective as in-person treatment.^103^

For patients with less severe symptoms, key recommendations to improve insomnia complaints include: (1) reducing time in bed; (2) get up at the same time every day; (3) do not go to bed unless sleepy; (4) do not stay in bed awake for more than 15–20 minutes; and (5) avoid napping. Table 1 includes examples of questions and associated behavior changes for each of these recommendations.

**TABLE 1.** Recommendations for Patients With *Subclinical* Insomnia, ie, Those Who Do Not Report Problems Falling Asleep, Staying Asleep, or Early Morning Awakening 3 or More Times per Week for At Least 3 Months

Question	Recommendation
*Problem: Excess time spent awake in bed* *Recommendation: Reduce time in bed*	
What do you do in bed before you try to go to sleep?	*If patient is using a screen (TV, phone, computer), eating, or reading in bed before going to sleep:* Could you do screen-based activity in your living room or kitchen earlier in the evening or before you get in bed? Using screen type right before trying to go to sleep likely wakes your brain up, making it harder to sleep. It also teaches your brain that the bed is a place for being awake, which worsens your sleep long term.
When you are having pain, what do you do and where do you spend time?	*If patient is spending time awake in bed in pain:* What about lying on the couch instead? Spending time in bed in pain is likely to worsen your sleep, as over time your brain will associate your bed with being awake and being in pain.
How many hours of sleep are you getting each night?	*If patient is sleeping less than 6.5 hours per night:* What limits your sleep? Are you interested in a referral to a person or program that could help? We generally know that sleeping less than 6.5 hours per night is associated with more health problems long term.
*Problem: Inconsistent sleep schedule* *Recommendation: Get up at the same time every day*	
Does your bed time or wake time change day to day? If so, by how much?	*If patient’s bedtime/wake time varies by more than 30 minutes one day to the next:* I’d like to recommend that you set your alarm for the same time every day, even on weekends. You’ll likely be tired at first, but after a few days it will likely improve your sleep, make it easier to keep the same schedule day to day, and help your body run better overall.
*Problem: Difficulty falling asleep* *Recommendation: Do not go to bed unless sleepy; avoid napping*	
How long does it take you to fall asleep at night?	*If patient is taking more than 30 minutes to fall asleep each night:* Would you consider getting in bed to go to sleep a little later, but still setting your alarm for the same time each morning? You’d be more likely to fall asleep quickly, which would be better for your sleep long term.
Do you worry or plan at night when you’re trying to fall asleep?	*If patient is worrying or planning in bed while trying to fall asleep:* Would you consider trying to either sort through your worries or calm down your mind before getting into bed? You could write down what’s on your mind a couple hours before bed or try a meditation or relaxation program, such as using the smartphone application “Mindfulness Coach.”
Do you take naps during the day? If so, how often?	*If yes and more than 2×/month:* When are you napping and for how long? Would you consider limiting your naps to 30 minutes and at least 8 hours before bedtime? This change would likely make it easier for you to fall asleep at night.
*Problem: Difficulty staying asleep* *Recommendation: Do not stay in bed awake for more than 15 minutes*	
Do you wake up a lot during the night? If so, how long are you spending awake each time?	*If patient is spending more than 30 minutes awake during their sleep cycle:* What wakes you up on these occasions? -If urination: would you consider limiting your fluid intake in the 3 hours before bedtime? You would likely wake up less often at night, leading to feeling more rested in the morning. -If related to ostomy bag/appliance: *discuss with patient, including providing education (eg, how full the bag should be before emptying) or problem-solving interfering symptoms that increase need for nighttime care (eg, gas).* When you’ve been awake for what feels like 15–20 minutes (not by watching the clock), get out of bed and do something boring (eg, folding laundry, reading a textbook, listening to mellow music) until you feel sleepy. Get out of bed at the same time you usually do, even if you have spent less time asleep that night. You’ll likely sleep better the next night.

Typical guidelines suggest that habitually taking more than 30 minutes to fall asleep and/or being awake in the middle of the night for more than 30 minute is often clinically significant.^104^ Importantly, adults with insomnia who are obtaining less than 6.5 hours of sleep per night via objective measurement have especially high morbidity rates for hypertension, cardiometabolic diseases, and diabetes.^105–107^ Insomnia, therefore, in addition to contributing to IBD morbidity, has broad health impact of other chronic diseases.

## CONCLUSIONS

Overall, the literature suggests that while pain, depression, and inflammation are not the primary targets for a course of CBT-I, they are all likely to improve significantly following treatment with CBT-I. This improvement is likely attributable to underlying physiological interactions between sleep, immune function, pain processing, and depressed mood.^108^ As insomnia and sleep disturbance are not only common in individuals with IBD, but also predict future symptom exacerbation, it is likely that CBT-I would be of benefit to this population. Further, as pain, depression, and inflammation are all common comorbidities and/or consequences of IBD, it is likely that patients treated with CBT-I would experience a number of widespread benefits.

Unfortunately, while the current literature supports the associations between sleep disturbances and IBD symptoms and comorbidities, there is presently no treatment research on sleep disturbances in IBD patients. Objective, prospective studies evaluating the impact of CBT-I on insomnia and IBD symptoms are warranted. Additionally, comprehensive evaluation of sleep disturbances in IBD using both self-report and objective measures would help to more accurately characterize sleep disturbances in these patients, thereby informing treatments.

## Data Availability

No new data were created or analyzed for the purposes of this manuscript.

## Notes

Funding: None.
